# Improvement in Frailty in a Patient With Severe Chronic Obstructive Pulmonary Disease After Ninjin'yoeito Therapy: A Case Report

**DOI:** 10.3389/fnut.2018.00071

**Published:** 2018-09-04

**Authors:** Hirai Kuniaki, Tanaka Akihiko, Homma Tetsuya, Mikuni Hatsuko, Kawahara Tomoko, Ohta Shin, Kusumoto Sojiro, Yamamoto Mayumi, Yamaguchi Fumihiro, Suzuki Shintaro, Ohnishi Tsukasa, Sagara Hironori

**Affiliations:** Division of Allergology and Respiratory Medicine, Department of Internal Medicine, Showa University School of Medicine, Tokyo, Japan

**Keywords:** frailty, chronic obstructive pulmonary disease, Ninjin'yoeito therapy, Kampo medicine, anorexia

## Abstract

Frailty is a poor prognostic factor in patients with chronic obstructive pulmonary disease (COPD). Although various studies have assessed the effects of conventional treatment with bronchodilators, nutritional support, and pulmonary rehabilitation for frailty in patients with COPD, none have addressed the effects of traditional Japanese medicine (Kampo medicine). Herein, we report the successful management of frailty using Ninjin'yoeito therapy in a 76-year-old patient with COPD. Despite being prescribed multiple bronchodilators, nutritional supplement therapy, patient education, and pulmonary rehabilitation, the patient exhibited unintentional weight loss, low energy, and low physical activity. Ninjin'yoeito was prescribed and these subjective symptoms began to improve 1 month after treatment initiation. In 6 months, the patient reported no frailty, had increased muscle mass, and had achieved an almost normal healthy state. Ninjin'yoeito has been associated with both physical effects, such as improvement in overall physical strength and appetite, and reduction in fatigue, and psychological effects, such as greater motivation and reduction of depression and anxiety symptoms. Physicians have usually treated COPD primarily with organ-specific treatments, such as bronchodilators; however, addressing both the physiological and psychological vulnerability has been difficult. This case report illustrates the potential usefulness of Ninjin'yoeito treatment for frailty in patients with COPD.

## Background

Frailty is defined as a non-specific state of physiological decline in advanced old age, characterized by vulnerability to adverse health issues, and it is a concept that has attracted the physicians' attention in patients with COPD. Many patients with COPD encounter frailty (1), and as frailty is expected to be reversible, particularly if addressed at an early stage, it is possible that the patients experience symptomatic improvement after appropriate interventions for correctable or removable factors (2). Various therapeutic methods, such as smoking cessation, pharmacologic therapy using bronchodilators, long term oxygen therapy, and pulmonary rehabilitation, showed efficacy in subsets of COPD comorbid patients (3). However, there are many patients with severe COPD who do not show improvement in frailty despite applying adequate treating strategies.

Traditional Japanese medicine (Kampo medicine) is known to be effective for several respiratory diseases (4–6), but to the best of our knowledge, only few studies have examined the effects of traditional Japanese medicine on frailty in patients with COPD. Ninjin'yoeito is a traditional Japanese medicine derived from 12 crude drugs, i.e., rehmannia root, Japanese angelica root, atractylodes rhizome, *Poria sclerotium*, ginseng, cinnamon bark, polygala root, peony root, citrus unshiu peel, astragalus root, glycyrrhiza, and schisandra fruit. Ninjin'yoeito prescription was approved by the Japanese Ministry of Health, and as it is used to treat fatigue, anorexia, night sweats, and anemia, we hypothesized that it may be beneficial for frailty. Herein, we have described a unique case of a patient who showed no improvement in frailty after conventional treatment but recuperated after Ninjin'yoeito administration.

## Case report

A 76-year-old man with severe chronic obstructive pulmonary disease (COPD) presented with a feeling of fatigue, weight loss, and reduced physical activities. He was diagnosed with COPD at the age of 69 years and had retired from work the following year. His smoking history included 40 cigarettes per day between the age of 14 and 69 years; his airflow limitation was classified as severe by the Global initiative for Chronic Obstructive Lung Disease; and a chest computed tomography (CT) scan showed severe emphysema. He had started long-term oxygen therapy at the age of 72 years and is currently inhaling 3 L/min of oxygen. Medical and family histories were otherwise unremarkable. Cardiac ultrasound excluded comorbid congestive heart failure or pulmonary hypertension, and CT pulmonary arteriography also excluded chronic pulmonary thromboembolism. As the patient had a history of acute exacerbations of COPD more than twice a year with extreme respiratory symptoms, he was prescribed a combination of inhaled long-acting antimuscarinic antagonist, long-acting beta2-agonist, corticosteroid, and oral carbocysteine, ambroxol, and theophylline. He reported symptoms of dyspnea on exertion, depression and anxiety, and a decrease in physical activity. He also experienced anorexia with a weight loss of more than 5 kg in a year, and no other possible causes of weight loss, such as tuberculosis and malignant tumor, were observed. Therefore, in addition to respiratory pharmacotherapy, we prescribed an antianxiety drug and provided nutritional supplement therapy, patient education, and pulmonary rehabilitation. However, the patient's mental and physical symptoms did not improve after 4 months. Furthermore, he exhibited deterioration in activities of daily living as well as physical and mental weakness; hospital visits were difficult and therefore, he considered home care. Persistent weight loss, poor endurance and energy, and low physical activity levels led to the diagnosis of physical frailty according to Fried's criteria (7). This vulnerability was supported by assessments using the Kihon Checklist (KCL) (8), the COPD Assessment Test (CAT) (9), and the Hospital Anxiety and Depression Scale (HADS) (10), all of which revealed high scores indicating inferior status. The KCL is a tool designed by a study group from the Japanese Ministry of Health, Labor and Welfare and comprises 25 items divided into seven categories: physical strength, nutritional status, oral function, socialization, memory, mood, and lifestyle. The KCL scores range from 0 (no frailty) to 25 (severe frailty); a previous study classified the patients' frailty status as non-frail (0–3), prefrail (4–7), and frail (8–25) (11). The CAT is a reliable tool that comprises eight items that assess the various COPD symptoms and is widely used in clinical practice. The CAT scores range from 0 to 40, with a score of 0 indicating no impairment. The HADS is also widely used to measure the level of anxiety and depression and comprises 14 items: 7 associated with anxiety (HADS-A) and 7 associated with depression (HADS-D). The HADS-A and HADS-D scores ranged from 0 to 21, and are in the range 8–10 for doubtful cases and ≥11 for definite cases. For the present case, we continued the pharmacological treatment, nutritional supplement therapy, patient education, and pulmonary rehabilitation and included 2.5 g of Ninjin'yoeito to be taken 3 times a day before meals.

**Figure 1:**
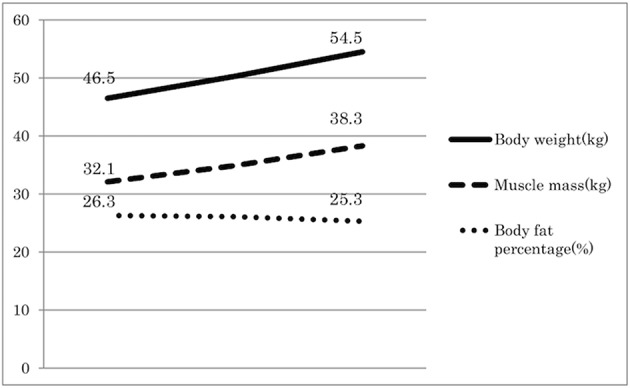
Body composition changes during Ninjin'yoeito therapy. Body composition was assessed at the indicated time points using bioelectrical impedance. Ninjin'yoeito administration increased the body weight and muscle mass without affecting body fat percentage.

**Figure 2:**
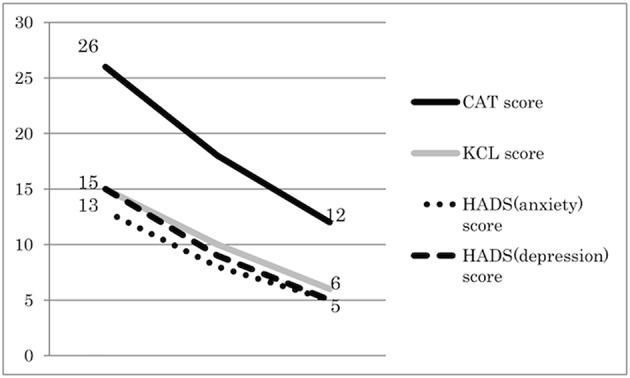
Changes in important factors of frailty and COPD during Ninjin'yoeito therapy. Ninjin'yoeito administration improved the patient's KCL, CAT, and HADS scores. The KCL comprises 25 items divided into seven categories: physical strength, nutritional status, oral function, socialization, memory, mood, and lifestyle. The CAT comprises 8 items that assess the various COPD symptoms. The HADS is used to measure the level of anxiety and depression and comprises 14 items.

After administration of Ninjin'yoeito, physical examination and blood tests such as electrolytes, liver function tests, and renal function test were performed to evaluate the side effects of Ninjin'yoeito administration. However, no side effects were detected. A significant improvement in symptoms, including increased appetite and alleviation of mood disorders and weight loss, was observed 1 month after initiating Ninjin'yoeito administration. Body weight and muscle mass continued to increase, and after 6 months of Ninjin'yoeito administration, the body weight increased by 8 kg compared with that prior to Ninjin'yoeito administration. Body composition assessed using bioelectrical impedance (InBody 720; Biospace, Tokyo, Japan) showed increasing muscle mass and no change in the body fat percentage (Figure 1). The patient's KCL, CAT, and HADS scores increased over time (Figure 2), and his status improved from frailty to non-frailty. Written informed consent was obtained from the participant for the publication of this case report.

## Discussion

This case report revealed two major findings. First, in patients who do not recover with conventional management of COPD, Ninjin'yoeito can improve their physical status from frailty to non-frailty. Second, Ninjin'yoeito can improve psychological frailty.

Ninjin'yoeito can improve physical frailty. The patient was categorized as having physical frailty because he fulfilled three of Fried's criteria, i.e., weight loss, feeling of fatigue, and decreased physical activity. All the three symptoms improved, and he could be categorized as non-frail at 6 months after initiating Ninjin'yoeito administration. COPD has a high morbidity and mortality rate, and various prognostic factors are being considered for COPD. Importantly, each item of Fried's criteria on the frailty tool has been shown to be a risk factor for COPD (12–14). Various factors, such as systemic inflammation, hypoxemia, increased respiratory effort, and hormonal abnormality, have been reported to result in appetite reduction and weight loss in patients with COPD (15). Although several intervention studies on weight loss have been reported (16–18), weight loss still remains refractory in some patients. In recent years, pulmonary rehabilitation has been demonstrated to improve frailty in patients with COPD; however, pulmonary rehabilitation in patients with severe frailty is more difficult to complete (19), and treatment strategies in frail patients with COPD who do not respond to conventional treatment are not clear. Our case report suggests that Ninjin'yoeito is a promising drug for frailty in patients with COPD who cannot complete pulmonary rehabilitation.

Ninjin'yoeito can improve the psychological frailty. Treatment of anxiety and depression in patients with COPD is particularly important, because it has been reported that the risk of ineffectiveness of conventional therapies increases if patients with COPD have psychological comorbidities (20, 21). The beneficial effects of Ninjin'yoeito were mirrored by favorable changes in the patient's KCL score, CAT score reflecting quality of life, and HADS score reflecting anxiety and depression. In support of our findings, previous studies have reported that Ninjin'yoeito can improve the mental status in patients with Alzheimer's disease (22).

Increase in physical activities has been shown to be an important factor in patients with COPD (14), and a reduction in both physical capacity (23) and mental health contributes to the decline in physical activity (21). We propose that the Ninjin'yoeito was effective in this case because it has multiple effects that address both physiological and psychological issues. Ninjin'yoeito is composed of 12 crude drugs each of which has its own unique effect(s). For example, ginseng and schisandra fruit have an anti-fatigue effect (24); ginseng and citrus unshiu peel have an anti-anorectic effect; and ginseng and atractylodes rhizome possess antidepressant and anti-aging effects. Thus, Ninjin'yoeito can be expected to improve the physical activity levels in patients with COPD because it positively affects both the physical and psychological symptoms. Additionally, the pulmonary pharmacological approach addresses only the organ-specific concerns rather than the comprehensive status of the patient, e.g., bronchodilators are prescribed to increase the airflow in patients with COPD. Recently, the concept of frailty, which comprehensively evaluates the state of a patient, is gaining attention. Furthermore, frailty is a particularly important risk factor in COPD, and there are no specific or effective management strategies. Thus, herein, it is important to note that while Ninjin'yoeito did not improve the respiratory function, it was effective in alleviating depression and anxiety symptoms and improving the appetite and physical activity levels, thereby acting as a “remedy for frailty.”

## Concluding remarks

This case report provides important preliminary evidence that Ninjin'yoeito therapy can improve frailty in patients with COPD. To the best of our knowledge, this is the first report of its kind. Future clinical studies are required to evaluate the effects of Ninjin'yoeito therapy on frailty in patients with COPD.

## Author contributions

HK, TA, OS, OT, and SH designed this study. HK, HT, YF, and KS performed clinical, functional, and laboratory assessments. SS, YM, MH, KT, and SH were involved in revising the manuscript. HK and TA wrote the manuscript. All authors significantly contributed to the data interpretation and manuscript preparation.

### Conflict of interest statement

The authors declare that the research was conducted in the absence of any commercial or financial relationships that could be construed as a potential conflict of interest.
